# Incidence and Paris Classification of Pediatric Inflammatory Bowel Disease

**DOI:** 10.1155/2014/904307

**Published:** 2014-03-20

**Authors:** Katalin Eszter Müller, Peter Laszlo Lakatos, Maria Papp, Gabor Veres

**Affiliations:** ^1^1st Department of Pediatrics, Semmelweis University, 53 Bókay Street, Budapest 1083, Hungary; ^2^1st Department of Medicine, Semmelweis University, Korányi S. Street 26A, Budapest 1083, Hungary; ^3^2nd Department of Medicine, University of Debrecen, Nagyerdei Körút 98, Debrecen 4032, Hungary

## Abstract

New epidemiological data suggest that the incidence of inflammatory bowel disease (IBD) is increasing. As a result the burden of disease accounts for more strains to the health care system. The clinical variability queries whether disease characteristics are related to clinical outcome. Our aim was to delineate the latest results of incidence trends in pediatric IBD and to compare the first experiences with Paris Classification. Incidence of pediatric IBD has been increasing in Western Europe and in Eastern Europe. To better characterize IBD, Paris Classification was introduced and validated recently. Ileocolonic involvement is the most characteristic disease location in Crohn's disease (CD) based on applying Paris Classification. The rate of perianal disease and complicated behaviour in CD was similar. It is of interest that CD patients with colonic involvement were less likely to have stricturing disease compared with patients with ileal involvement. In addition, pancolitis dominated in ulcerative colitis (UC). However, most countries lack prospective, nationwide epidemiological studies to estimate incidence trends. This review emphasizes the importance of nationwide registries that enroll all pediatric IBD cases serving reliable data for “everyday practice.” These first reports have shown that Paris Classification is a useful tool to determine the pediatric IBD phenotype.

## 1. Introduction

The inflammatory bowel diseases (IBD), Crohn's disease (CD), and ulcerative colitis (UC) are chronic inflammatory disorders of the gastrointestinal tract of unknown etiology. It is hypothesized that IBD is due to a dysregulated mucosal immune response to commensal gut flora in genetically susceptible individuals. However, causes of the dysregulated immune response are not delineated. The fluctuating disease course affects quality of life in IBD patients significantly. Furthermore, CD and UC account for substantial costs to the health care system and society [1]. New epidemiological data suggest that the incidence and prevalence of IBD are increasing. In addition, medical therapy and disease management have changed significantly in the last decade. IBD develops during childhood or adolescence in up to 25% of IBD patients. According to a recent report from the USA the total hospital charges for pediatric CD increased significantly from $81 million in 1997 to $194 million in 2009. Similarly, total hospital charges for UC rose from $53 million in 1997 to $143 million in 2009 [2].

The clinical presentation of CD and UC is highly variable, with significant diversity in phenotypes of the diseases [11]. This diversity in adults is specified by differences in the location the natural history and the outcomes. The clinical heterogeneity raised the question whether disease characteristics (age, location, and behaviour) had been related to clinical outcome. As a result Vienna and Montreal Classifications have been processed to classify IBD using clinical and epidemiological features. Patients are classified based on phenotypic characteristics (age, location, and behaviour). Oostenbrug et al. analyzed disease characteristics of 292 adult CD patients according to Vienna classification. The operation rate was higher in patients with ileocolonic localization (*P* < 0.05) and stricturing and penetrating disease behaviour (*P* < 0.001) [12], confirming that disease and epidemiological characteristics are associated with outcome in CD.

Previous epidemiological studies reported marked differences in pediatric- and adult-onset IBD. Nevertheless, previous classification systems (e.g., Montreal Classification) were not designed or validated for pediatric patients. The Paris Classification is a new evidence-based consensus recommendation for pediatric modification of the Montreal criteria [13]. So far, this new classification system has only been applied in a few population-based studies.

In this report we summarize the latest incidence trends of pediatric IBD and the first experiences with Paris Classification.

## 2. Incidence of Adult-Onset Inflammatory Bowel Disease and Geographic Distribution

Crohn et al. published a case series with ileitis terminalis in 1932 where the “terminal” word, contrary to the popular belief, indicated not the location (terminal ileum) but the “deadly, terminal disease” [14]. The increasing incidence of IBD was observed from the middle of the 20th century. According to the first epidemiological studies the incidence of IBD differed greatly in geographical areas. The frequency of IBD was higher in developed countries than in developing countries and higher in northern areas than in southern regions (Table 1) [15, 16].

Of note, according to the recent studies the traditional geographical distribution of IBD has changed in the last 20 years. In most Western countries the incidence of adult UC and CD has stabilized, while incidence has been rising in regions with previously low incidence (Southern and Eastern Europe and Asia). In the latest systemic review about the changes in the worldwide incidence of IBD the highest incidence of adult UC was 24.3/10^5^ person-years in Europe, 19.2/10^5^ in North America, and 6.3/10^5^ in Asia and the Middle East. The highest incidence of adult CD was 20.2/10^5^ in North America, 12.7/10^5^ in Europe, and 5.0/10^5^ in Asia and the Middle East. Furthermore, a statistically significant increase in incidence of IBD was observed in 75% of CD studies and 60% of UC studies [17]. Extrapolation of the incidence figures on the total European population indicates around 78.000 new cases of CD and 178.000 of UC yearly [1]. The prevalence of CD may be up to 1.6 million people with CD and 2.1 million people with UC in Europe if reported prevalence rates are extrapolated for the total European population.

Some studies suggested that the incidence of IBD decreases from the north to the south comparing incidence within some countries and between countries. The incidence of UC in Copenhagen, Denmark (8.1/10^5^), was four times higher than in Bologna, Italy (1.9/10^5^) [18]. For CD, the rate of 4.1/10^5^ in Copenhagen was five times higher than in Galicia, North-West Spain (0–8/10^5^). To establish the north-south gradient in Europe a prospective study (EC-IBD) was conducted in 20 centers that focused on frequency of IBD across the continent. According to the EC-IBD study rates of UC in northern centers were 40% higher than those in the south (11.4/10^5^ versus 8.9/10^5^) [18]. It was concluded that the excess is less than expected on the basis of previous studies that may suggest an increase in the incidence of IBD in Southern Europe whereas those in the north may have reached a plateau. However, some recent studies still show significant difference in frequency of IBD within countries in children and in adults [6, 7, 19, 20]. The etiology of the north-south gradient is not delineated. The latest hypothesis suggests that level of vitamin D is lower in populations living in Northern Europe. Vitamin D is known to be an inducer of NOD2 function and the lack of vitamin D may result in the lower activity of NOD2 and this factor may contribute to the higher incidence of CD in regions with low sunrise exposure [21, 22].

A few population-based cohort data have been reported from Eastern Europe showing an increase in previously less industrialized countries recently [23]. The elevating rates of IBD in these countries could be due to methodological bias rising awareness of the disease and improved availability of diagnostic tools. Consequently, a prospective, population-based cohort study (EpiCom study) was established to investigate whether there is an east-west gradient in the incidence of IBD in Europe. Thirty-one centers participated across Europe and this cohort of IBD patients covers a background population of 10.1 million people. The overall annual incidence rates in all Western European centers were roughly twice as high as rates in all Eastern European centers for CD (incidence rate ratio (IRR = 1.9)) and UC (IRR = 2.1). The diagnostic approach for CD and UC seemed similar in Eastern and Western Europe. It is of interest that the incidences correlated with the GDP of each country [24]. The difference in incidence between Eastern and Western Europe and the rapid increase in incidence rates in Eastern Europe support the role for environmental factors. In the past two decades there has been a change in the lifestyle in Eastern Europe, including the diet (western lifestyle). Due to westernized diet (“junk food,” food additives) luminal antigen exposure has been changed in this region. This alteration may be an important trigger in the pathogenesis of IBD [23].

Briefly, the incidence of IBD is rising with time around the world, indicating its emergence as a global disease [17]. The heterogeneity of IBD frequency in different regions highlights the role of environmental factors.

## 3. Incidence of Pediatric-Onset IBD

**Figure 1:**
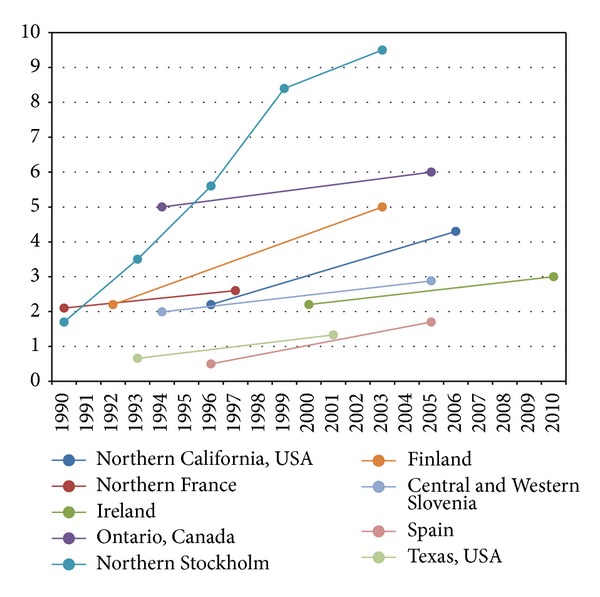
Incidence trends in pediatric Crohn's disease from 1990 to 2010 [5, 7, 32, 34–39].

The majority of pediatric population-based reports before 1990s showed that the incidence of pediatric IBD is rising and the frequency of CD is higher than that of UC (Figure 1) [25–27]. Some studies mainly from Northern Europe described the dominance of UC [28, 29]. Incidence of pediatric IBD has been also rising according to the registry of Veszprem Province (Hungary). In CD, incidence increased from 0 in 1977–1981 to 7.2/10^5^ in 2007–2011. The incidence of pediatric UC increased from 0.7/10^5^ in 1977–1981 to 5.2/10^5^ in 2007–2011 [30].

**Figure 2:**
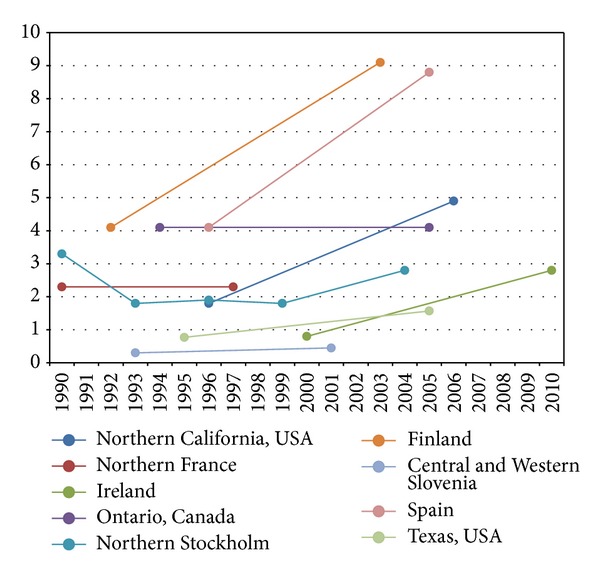
Incidence trends in pediatric Crohn's disease from 1990 to 2010 [5, 7, 32, 34–39].

Temporal trends in the incidence of pediatric-onset IBD were controversial until recently (Figures 1 and 2). A systematic review describing the epidemiology of childhood-onset IBD was conducted to evaluate the alteration in incidence over the last decades. Articles published during 1950–2009 were searched and analyzed. Statistical trends in incidence of pediatric IBD were tested in nine publications; seven (77.8%) reported increased incidence over time. Twenty-five studies calculated temporal trends in CD incidence, and 15 (60.0%) described a significant increase. Twenty studies analyzed temporal trends of incidence in UC. Four (20.0%) of them reported significant increase; however, 13 reports (65.0%) showed no significant change. Rising rates of pediatric IBD were observed in both developed and developing nations [31].

Recently a few new epidemiological pediatric cohort studies revealed an increasing trend in incidence of pediatric IBD. Hope et al. investigated the incidence rate of pediatric IBD between 2000 and 2010 in Ireland. Incidence of IBD was 2.5/10^5^ in 2000 that elevated to 5.6/10^5^ by 2010. The mean annual increase in CD incidence was 0.153 (*P* = 0.04), and for UC it was 0.175 (*P* < 0.01) between 2000 and 2010 [32]. Geographically close to Ireland a Scottish report found a similar rising trend. A national cohort of prospectively and retrospectively acquired incident cases of pediatric IBD diagnosed less than 16 years old in pediatric services in Scotland was captured for the period 2003–2008. The incidence of CD was 4.75/10^5^, and incidence rate of UC was 2.06/10^5^. Significant increase in the incidence of IBD (from 4.45/10^5^, *P* < 0.0001), CD (from 2.86/10^5^, *P* < 0.0001), and UC (from 1.59/10^5^, *P* = 0.023) was found compared with data from 1990 to 1995 [33].

The sex- and age-standardized incidence of pediatric IBD in Northern Stockholm during 2002–2007 was 12.8/10^5^ for IBD, 9.2/10^5^ for CD, and 2.8/10^5^ for UC. An increasing incidence rate of UC (58.4%, *P* < 0.01) was observed during the study period. However, temporal trend for the incidence of IBD (3.2%, *P* = 0.54) was not remarkable. Meanwhile, the incidence rate of pediatric IBD in Northern Stockholm was significantly higher in 2002–2007 than that published in earlier study covering 1990–2001. The former sharp increase in incidence of pediatric CD seems to reach a plateau, although at a higher rate than reported from most other regions in the world [34]. Another Scandinavian report found similar trends. In Eastern Denmark the frequencies of UC and CD have traditionally been similar [40]. The mean annual incidence rates in a prospective cohort during 2007–2009 were 6.4/10^5^ for IBD, 3.2/10^5^ for CD, and 3.1/10^5^ for UC. The mean incidence rates of IBD over the past 12 years (1998–2009) in Eastern Denmark increased significantly (trend test, *P* = 0.02).

The latest report from Southern Europe was conducted in Spain. A retrospective survey of patients diagnosed below 18 years of age in the period 1996–2009 was performed. Patients' data were obtained from the hospitals' databases. Martin-de-Carpi et al. described also a rise in IBD from 0.97/10^5^ to 2.8/10^5^ during 1996–2009. Although this increase is more evident for CD (from 0.53/10^5^ to 1.7/10^5^), UC has also risen (from 0.39/10^5^ to 0.88/10^5^) [7].

An east-west gradient has been reported in adults, as previously mentioned. A recent study from Eastern Europe has been published from the north-eastern part of Slovenia. The mean annual incidence was 7.6/10^5^ for IBD, 4.6/10^5^ for CD, and 2.8/10^5^ for UC. The incidence of total IBD, CD, and UC increased from 5.7/10^5^, 3.9/10^5^, and 1.8/10^5^ in the period 2002–2004, respectively, to 8.9/10^5^, 5.0/10^5^, and 3.4/10^5^ in the period 2008–2010. Data of our prospective Hungarian Pediatric IBD Registry (HUPIR) are comparable to the rate of Slovenia. The mean annual incidence rate of pediatric IBD was 7.48/10^5^, for CD it was 4.72/10^5^, and for UC it was 2.32/10^5^ during 2007–2009 in Hungary [3]. The incidence of childhood IBD in Eastern Europe seems to be high and comparable to that in western countries of Europe.

In conclusion, incidence rate of pediatric IBD has been increasing in Northern and in Southern Europe as well as in Eastern Europe.

## 4. Methodological Problems in Assessing Incidence of IBD in Different Regions

Incidence rates reported in different studies are not always directly comparable due to heterogeneity in study design and diagnostic criteria, and these factors can dramatically affect the incidence rates. A key issue is that some studies use hospital records while others use surveys and administrative data. The age limit is an important inclusion criterion with great impact on incidence rate. Most data are retrospective, only a few prospective population-based studies were conducted, especially reports from Asia or Eastern Europe [4, 41]. Furthermore, the incidence rates are often an extrapolation from one or more regions of a country; however, some studies reported regional variation in frequency of IBD using consistent methodologies, implying that different regions within a country may have different incidence rates [31]. In summary, most countries lack accurate estimates of incidence of pediatric IBD.

## 5. Reasons for Rising Incidence

The cause of increasing incidence is not established. Rising incidence seems to be true both in children and adults, suggesting that the apparent increases in IBD incidence are genuine [42]. Twin studies have shown 16%–36% concordance rates in monozygotic twins and 4% concordance rates in dizygotic twins suggesting that genetic risk factors have some role in the pathogenesis of IBD [31]. As a result, in the absence of large genetic background shifts, changing rates of IBD incidence highlight the importance of environmental factors.

The socioeconomic alteration in previously low-incident areas “from developing to developed” may be related to this rising occurrence. The spread of western lifestyle seems to be related to the elevation in incidence of Eastern European countries and in Asian countries. In concordance, emigrants of South Asian origin emigrating to Canada and UK have been observed to have an increased risk for IBD, confirming that environmental factors contribute to this higher risk [43].

The “cold chain hypothesis” suggested that refrigeration may have altered the bacterial content of our diet, resulting in the increased growth of disease-triggering organisms [44]. The well-known “hygiene hypothesis” suggests that a cleaner environment, smaller families, and lower exposure to farm animals have resulted in increased risk of IBD in westernized nations [45]. Furthermore, perinatal and early life events may also play a significant role in developing IBD [46].

## 6. Incidence Rates in Childhood in Different Age Groups

The rising incidence could be the consequence of the shift towards onset at a younger age; however this increase is evident in adult [17] and in pediatric-onset IBD [31] as well [47]. Incidence rate by age groups in childhood could also bring up further questions. Only a few studies reported trends of incidence after stratifying the children. Two studies divided patients into three age groups (0–5 years, 6–10 years, and 11–15 years). Henderson et al. compared incidence rates of two periods (1990–1995 and 2003–2008) and found that there was a significant increase in incidence of patients between 11 and 15 years (from 7.8/10^5^ to 11.8/10^5^, *P* = 0.052) and patients between 6 and 10 years (from 4.3/10^5^ to 7.4/10^5^, *P* = 0.039). Frequency of IBD also increased in children younger than 5 years, but this trend was not significant (from 0.9/10^5^ to 1.5/10^5^, *P* = 0.292) [33]. This result is similar to the report from Texas [35]. Incidence of IBD (compared in periods of 1990–1996 and 1997–2002) increased significantly in age groups 10–14 years and 15–17 years. However, in children between 5 and 9 years the rise was not significant and incidence was stable in children under five. Findings of a report from California are comparable; the rising trend of UC over time was significant for the age group 10 to 14 years (comparing periods of 1996–1999, 2000–2002, and 2003–2006), but the trend in children aged 15 to 17 years was not significant [36]. Chouraki et al. stratified patients into two age groups (0–9 years and 10–19 years) and also reported an obvious increase of IBD in older children and a slight increase in younger children comparing incidence rate of IBD in 1988–1990 and in 2006-2007 [6]. In contrast with these findings, Jakobsen et al. did not observe difference in incidence rates over a 12-year period (1998–2009) after stratifying the patients into three 5-year age groups (0–4 years, 5–9 years, and 10–14 years) [48]. The only report describing a significantly rising incidence of IBD patients younger than 5 years came from Ontario, Canada [37].

Summarizing these data, only a few studies investigated long term changes of incidence in children stratified by age. According to these data, incidence is clearly increasing in children older than 10 years. In contrast, this trend is not obvious in children younger than 5 years, suggesting that this subgroup of patients is unique. One explanation could be that IBD in younger children is more likely to be genetically determined, and environmental factors may contribute to lesser extent to the pathogenesis of intestinal inflammation. However, in older children increasing incidence by age highlights the dominant role of environmental triggers. In conclusion, these results suggest that the rising incidence occurs mainly in children older than 10 years which could indicate the importance of environmental factors.

## 7. First Experiences with Paris Classification of Patients with Pediatric-Onset IBD

IBD develops during childhood or adolescence in up to 25% of patients. Pediatric IBD differs in some clinical characteristics from adult IBD. As previously mentioned, CD is more frequent in children than UC in contrast to adults. A male genome-wide association dominance has been observed in children with CD, while females are more frequently affected in adulthood. Despite higher familial occurrence of IBD in children genomewide association studies showed that multiple genes conferring susceptibility are comparable [49, 50]. A key feature of pediatric-onset IBD is the potential impaired growth retardation and delayed puberty. According to previous studies comparing IBD in children and adults [48, 51, 52], ileocecal location is more common in adults than in children, while panenteric disease is characteristic phenomenon in pediatric-onset CD. It is of interest that there is an association between pediatric CD patients carrying one of the three NOD2 mutations and ileocolonic involvement [53]. However, younger children with CD, similar to older adults and the elderly, are more likely to have colonic disease [54, 55]. Furthermore, upper gastrointestinal involvement is more common in pediatric IBD (16–51%). This wide range is due to two methodical approaches. On one hand, there is a difference in routine diagnostic procedure in adults and children, since workup of pediatric IBD includes gastroscopy, ileocolonoscopy, and small bowel imaging [56]; meanwhile adult gastroenterologists perform usually ileocolonoscopy and radiology. This routine may lead to underestimation of upper gastrointestinal involvement in adult CD patients. On the other hand, disease involvement was defined as ulceration or aphthous ulcers in Vienna Classification. However, in several pediatric reports, microscopic involvement has been applied as a diagnostic criterion. There is no consensus at present about what abnormalities should be regarded as proof of involvement in upper gastrointestinal biopsies. Thus, many nonspecific findings on gastroduodenal biopsies may be interpreted as evidence of disease involvement in this region [11]. In UC there is also clear difference in disease location between children and adults. Pediatric-onset UC patients are more likely to have pancolitis (60–70%); however, 20–30% of adults present with proctitis.

Due to weaknesses of Montreal Classification with regard to pediatric IBD, a modified classification (Paris) has been devised [13]. The new Paris Classification included classifying age at diagnosis as A1a (0 to <10 years), A1b (10 to <17 years), A2 (17 to 40 years), and A3 (>40 years), distinguishing disease above the distal ileum as L4a (proximal to ligament of Treitz) and L4b (ligament of Treitz to above distal ileum), allowing both stenosing and penetrating disease to be classified in the same patient (B2B3), denoting the presence of growth failure in the patient at any time as G1 versus G0 (never growth failure), adding E4 to denote extent of ulcerative colitis that is proximal to the hepatic flexure, and denoting ever severe ulcerative colitis during disease course by S1 (Tables 2 and 3).

### 7.1. Newly Diagnosed Pediatric IBD Patients According to Paris Classification

Recently a few center- and population-based studies analyzed newly diagnosed pediatric IBD patients according to Paris Classification: (1) a study of prospectively collected IBD patients younger than 15 years diagnosed in Northern Stockholm (2002–2007) [34]; (2) a retrospective study of IBD patients younger than 16 years from Ireland; (3) the Eurokids registry, a prospective, center-based registry of newly diagnosed pediatric IBD patients in 44 IBD centers in 18 countries [57]; (4) a retrospective study on a cohort of newly diagnosed children aged 0–18 years from North-Eastern Slovenia (2002–2010) [58]; (5) our prospective, nationwide, incident cohort of pediatric IBD patients younger than 18 years from Hungary [3] (Tables 4 and 5). The main findings were similar to earlier reports and were comparable to each other: (1) ileocolonic involvement was the characteristic disease location in CD; (2) pancolitis dominated in UC; (3) rate of perianal disease and complicated behaviour was similar.

However, the reports from Northern Stockholm and Ireland showed a higher rate of pure colonic CD than the others (71% and 45% versus 27–28%), which is in contrast with most population-based studies from both Europe and North America [40, 59, 60]. Among earlier studies two reports described similar figures of isolated colonic CD (Scotland 66% [51]) and Sweden 43% [61]). Further studies are needed to determine if these contrasts point to possible disease-modifying environmental or other factors. However, all of these studies applied an age limit of 16 years, whereas the age limit of studies with lower rate of isolated colonic involvement included patients younger than 18 years. This difference in inclusion criteria may contribute to a shift of proportion of patients with purely colonic CD.

### 7.2. Follow-Up and Paris Classification

Hope et al. followed up Irish IBD patients for 2 years and found that the progression of disease extension in CD during the first 2 years of disease course was not frequent [32]. This result is in contrast with the disease extension presented by van Limbergen et al. (39% at 2 years) [51] and by Vernier-Massouille et al. (31% at median follow-up of 84 months) [60]. Analysis of the 196 childhood-onset CD patients demonstrated that 53 of 196 (27.0%) had panenteric involvement (L3 + L4) at diagnosis [51]. During 2 years follow-up, 56 (39.1%) children had progression in disease extension: changes were mostly due to extension from localized disease to more extensive disease involving the lower gastrointestinal tract (41/56, 73.2%). Meanwhile, the proportions of disease location in UC had not changed significantly [51] at last follow-up. The conflicting results may be due to methodological differences (longer recruitment and follow-up period in earlier studies). However, the location of disease changed over time in other publications conducted in adults as well [62]. This may query the relationship of disease course and initial disease characteristics.

In the report from North-Eastern Slovenia progression of disease extension was also investigated. At diagnosis, 16.3% had panenteric disease (L3L4ab), while extensive involvement was observed in 21.6% of patients during the follow-up period [58]. In UC, proportion of patients with pancolitis (E4) increased from 61.5% to 76.5%, respectively. At presentation, only 6% of CD patients had stricturing and 8% had penetrating phenotype, and these complicated phenotypes doubled during the follow-up. In agreement with these figures other studies also observed a similar 2-fold rise in complicated CD behaviour during the follow-up [32, 51, 60]. In UC, rate of patients with pancolitis (E4) increased from 61.4% to 76.5%.

### 7.3. Paris Classification and Clinical Characteristics

Association of epidemiological and disease characteristics, like family history and extraintestinal manifestations (EIM), with phenotype according to Paris Classification has been analyzed in two recent studies. In our study we did not find any significant relationship between age and gender distribution or family history, EIM, and disease location in CD or in UC [3]. In contrast, de Bie et al. described that isolated colonic disease was recorded in 41% (47/114) of children diagnosed before 10 years of age compared with 24% (111/467) of older children (*P* < 0.001) [57]. A similar trend was found in the Hungarian cohort, though the difference was not significant. The two studies concurred that upper gastrointestinal involvement was not related to age, gender, family history of IBD, or presence of EIM. However, Lazarev et al. described a significantly greater risk for multiple abdominal surgeries in patients with jejunal involvement than in patients with upper gastrointestinal involvement proximal to the ligament of Treitz [63].

According to Eurokids, rate of perianal disease occurred more often in patients with B3 than in patients with B1 (38% versus 8%, *P* < 0.001) and B2 (38% versus 7%, *P* < 0.001). In addition, patients with L2 disease were less likely to have stricturing disease complications compared with patients with L1 or L3 disease (6% versus 21% versus 15%, *P* = 0.005). These latter results were not observed in our study, which is may be due to the lower number of included patients (582 versus 247). This discrepancy is probably due to the different population of the two studies. Eurokids registry is not a population-based cohort, but a selection of centers with special interest in IBD; meanwhile the HUPIR is a population-based incident cohort involving less severe cases. This phenomenon emphasizes the importance of nationwide registries that enroll all pediatric patients with IBD including less severe cases also.

In conclusion, the first reports have shown that Paris Classification is a useful tool to determine the characteristic pediatric CD phenotype. Location of disease is comparable in studies with Paris Classification to studies classifying patients according to earlier classification systems.

## 8. Conclusions

The worldwide increasing trends of IBD incidence seem to be evident in adults [17] as well as in children [31]. Exploring increase in incidence of IBD may provide useful insights into the pathogenesis, especially with regard to environmental factors related to industrialization, such as changes in hygiene, a more westernized diet, economic growth, and the shift from rural to urban environments [42]. Furthermore, clinical classification, like Paris Classification, may contribute to define distinct subgroup of patients with different prognosis with different therapeutical approach.

However, there are several methodological clues that complicate comparing studies from different regions. Consequently, more prospective, population-based studies (data should not only come from center specialized for IBD) are needed to delineate the frequency of IBD and disease phenotype.

## Figures and Tables

**Table 1:** Incidence rates of pediatric- and adult-onset Crohn's disease and ulcerative colitis in different countries (/100,000).

	Pediatric Crohn's disease	Adult Crohn's disease	Pediatric ulcerative colitis	Adult ulcerative colitis
Hungary (2007–2009/2002–2006) [3, 4]	4.8	8.9	2.1	11.9
Northern France (1988–1998/2006-2007) [5, 6]	2.3	6.7	0.8	3.4
Nationwide/Madrid, Spain (2003–2007/2003–2005) [7, 8]	1.7	7.3	0.88	7.1
Copenhagen County, Denmark (2002–2004/2003–2005) [9, 10]	3.1	8.6	2.7	13.4

**Table 2:** Comparison of Montreal and Paris Classifications for Crohn's disease based on Levine et al. [13].

	Montreal Classification	Paris Classification
Age at diagnosis	A1: below 17 years A2: 17–40 years A3: above 40 years	A1a: 0–<10years A1b: 10–<17 years A2: 17–40 years A3: >40 years

Location	L1: terminal ileal/ limited cecal disease L2: colonic L3: ileocolonic L4*: isolated upper disease	L1: distal 1/3 ileum 6 limited cecal disease L2: colonic L3: ileocolonic L4a: upper disease proximal to ligament of Treitz* L4b: upper disease distal to ligament of Treitz and proximal to distal 1/3 ileum*

Behaviour	B1: nonstricturing nonpenetrating B2: stricturing B3: penetrating p: perianal disease modifier	B1: nonstricturing nonpenetrating B2: stricturing B3: penetrating B2B3: both penetrating and stricturing disease, either at the same or different times p: perianal disease modifier

Growth	—	G0: no evidence of growth delay G1: growth delay

*In both the Montreal and Paris Classification systems L4 and L4a/L4b may coexist with L1, L2, and L3, respectively.

**Table 3:** Comparison of Montreal and Paris Classifications for ulcerative colitis based on Levine et al. [13].

	Montreal Classification	Paris Classification
Extent	E1: ulcerative proctitis E2: left-sided UC (distal to splenic flexure) E3: extensive (proximal to splenic flexure)	E1: ulcerative proctitis E2: left-sided UC (distal to splenic flexure) E3: extensive (hepatic flexure distally) E4: pancolitis (proximal to hepatic flexure)

Severity	S0: clinical remission S1: mild UC S2: moderate UC S3: severe UC	S0: never severe* S1: ever severe*

*Severe defined by Pediatric Ulcerative Colitis Activity Index (PUCAI).

**Table 4:** Paris Classification of patients with Crohn's disease in population-based studies in Europe [3, 32, 34, 57, 58].

	North-Eastern Slovenia	Northern Stockholm	Eurokids	Hungary (HUPIR)	Ireland
Crohn's disease (*n*)	43	96	582	247	31
Age, % (*n*/*n*)					
A1a	15	—	20% (244/1221)	11% (27/247)	26% (8/31)
A1b	—	—	80%	78% (197/247)	74% (23/31)
A2	—	—		9% (23/247)	
Location, % (*n*/*n*)					
L1*	20.9% (9/43)	8% (8/96)	16%	13.4% (33/247)	19% (6/31)
L1 + L4a	2.3%	—	3.6% (21)		
L1 + L4b	0	—	3.4% (20)	3% (7/247)	13% (4/31)
L1 + L4ab	7%	—	1.4% (8)		
*L2	4.6% (2/43)	71% (68/96)	28% (159/582)	27.5% (68/247)	45% (14/31)
L2 + L4a	0	—	4.1% (24/582)		
L2 + L4b	0	—	3.8% (22/582)	6.8% (17/247)	3% (1/31)
L2 + L4ab	0	—	1.2% (7/582)		
*L3	74.5% (32/43)	20% (19/96)	53%	58.7% (145/247)	32% (10/31)
L3 + L4a	23.3%	—	14.3%		
L3 + L4b	11.6%	—	6.5%	49	16% (5/31)
L3L4ab	16.3%	—	4.3%		
L4 (Isolated)	0	0	4% (18/582)	0.4% (1/247)	3% (1/31)
All upper gastrointestinal involvement				0.4% (1/247)	
L4a	48.9% (21/43)	17% (16/96)			
L4b	34.9% (15/43)	1% (1/96)			
Behaviour					
B1	86% (56/65)	95% (91/96)	82% (959/1177)	12.1% (216/256)	90% (28/31)
B2	6% (4/65)	5% (5)	12% (144/1177)	2.3% (31/256)	6% (2/31)
B3	8% (5/65)	0	5% (55/1177)	1.2% (6/256)	3% (1/31)
B2B3	—	0	2% (19/1177)	0.6% (3/256)	—
Perianal disease	—	8% (8/96)	9% (114/1207)	14.5% (37/247)	10% (3/31)
Growth (G1) G1				6.6% (16/244)	23% (4/31)

*L1 + L4a, L1 + L4b, and L1 + L4ab patients are included in patients with L1 location.

A1a: 0–<10 years, A1b: 10–<17 years, and A2: 17–<40 years. B1: nonstricturing-nonpenetrating; B2: stricturing; B3: penetrating; B2B3: both penetrating and stricturing; G1: evidence of growth delay; L1: distal 1/3 ileal disease (limited cecal disease); L2: colonic disease; L3: ileocolonic disease; L4: upper gastrointestinal tract disease; L4a: esophagogastroduodenal disease proximal to ligament of Treitz; L4b: distal to ligament of Treitz.

**Table 5:** Paris Classification of ulcerative colitis patients in population-based studies in Europe [3, 32, 34, 57, 58].

	North-Eastern Slovenia	Northern Stockholm	Eurokids	Hungary (HUPIR)	Ireland
Ulcerative colitis (*n*)	39	29	578	121	14
E1	5.2% (2/39)	11% (3/29)	5% (27/578)	5% (6/121)	14% (2/14)
E2	25.6% (10/39)	14% (4/29)	18% (104/578)	24.8% (30/121)	14% (2/14)
E3	7.7% (3/39)	4% (1/29)	9% (50/578)	13.2% (16/121)	7% (1/14)
E4	61.4% (24/39)	75% (21/29)	69% (397/578)	57% (69/121)	65% (9/14)
Severity (S1)	—	—	—	18.6% (13/121)	43% (6/31)

E1: ulcerative proctitis; E2: left-sided ulcerative colitis (distal to splenic flexure); E3: extensive colitis (hepatic flexure distally); E4: pancolitis (proximal to hepatic flexure); S1: severe at some stage.
